# Assessing Bacterial Interactions Using Carbohydrate-Based Microarrays

**DOI:** 10.3390/microarrays4040690

**Published:** 2015-12-10

**Authors:** Andrea Flannery, Jared Q. Gerlach, Lokesh Joshi, Michelle Kilcoyne

**Affiliations:** 1 Carbohydrate Signalling Group, Microbiology, School of Natural Sciences, National University of Ireland Galway, Galway, Ireland; E-Mail: a.flannery5@nuigalway.ie; 2 Advanced Glycoscience Research Cluster, National Centre for Biomedical Engineering Science, National University of Ireland Galway, Galway, Ireland; E-Mail: jared.gerlach@nuigalway.ie; 3 Regenerative Medicine Institute, National University of Ireland Galway, Galway, Ireland; 4 Glycoscience Group, National Centre for Biomedical Engineering Science, National University of Ireland Galway, Galway, Ireland; E-Mail: lokesh.joshi@nuigalway.ie

**Keywords:** bacteria, carbohydrate microarrays, glycan microarrays, adhesins, mucins, bacterial polysaccharides, cross-talk, microorganisms, polysaccharides, glycomics

## Abstract

Carbohydrates play a crucial role in host-microorganism interactions and many host glycoconjugates are receptors or co-receptors for microbial binding. Host glycosylation varies with species and location in the body, and this contributes to species specificity and tropism of commensal and pathogenic bacteria. Additionally, bacterial glycosylation is often the first bacterial molecular species encountered and responded to by the host system. Accordingly, characterising and identifying the exact structures involved in these critical interactions is an important priority in deciphering microbial pathogenesis. Carbohydrate-based microarray platforms have been an underused tool for screening bacterial interactions with specific carbohydrate structures, but they are growing in popularity in recent years. In this review, we discuss carbohydrate-based microarrays that have been profiled with whole bacteria, recombinantly expressed adhesins or serum antibodies. Three main types of carbohydrate-based microarray platform are considered; (i) conventional carbohydrate or glycan microarrays; (ii) whole mucin microarrays; and (iii) microarrays constructed from bacterial polysaccharides or their components. Determining the nature of the interactions between bacteria and host can help clarify the molecular mechanisms of carbohydrate-mediated interactions in microbial pathogenesis, infectious disease and host immune response and may lead to new strategies to boost therapeutic treatments.

## 1. Introduction

Carbohydrates play a crucial role in a wide variety of biological processes such as cell-cell recognition, metastasis, immune system mediation and function, intracellular trafficking and progression of many diseases including cancer [1,2]. They are also critically important in host-microorganism interactions and many host glycoconjugates are receptors or co-receptors for microbial binding [3]. Host glycosylation varies with species and location in the body and this contributes to species specificity and tropism of commensal and pathogenic bacteria [4]. Additionally, bacterial glycosylation is often the first bacterial molecular species encountered, recognised and responded to by the host system. Bacterial polysaccharides and other glycoconjugates are usually antigenic and when these antigens mimic host carbohydrate structures to evade immune detection, autoimmune disorders can result [3,5]. Accordingly, characterising and identifying the exact structures involved in these critical interactions can lead to a better understanding of microbial pathogenesis and the mechanisms of infectious disease.

**Figure 1:**
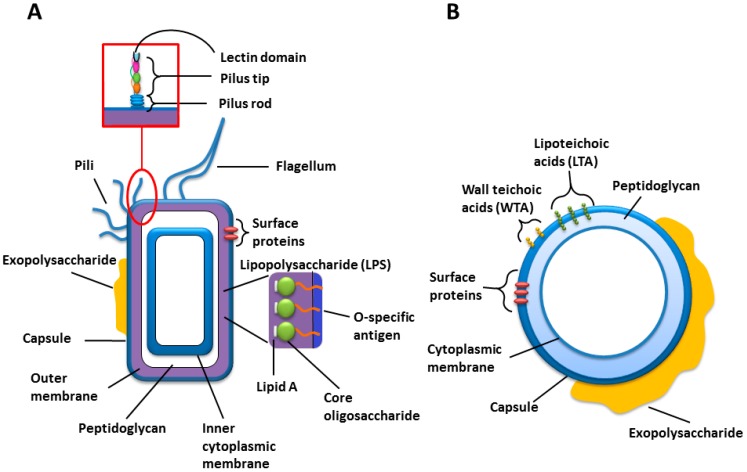
Cartoons of the general cell wall structures and components of (**A**) Gram-negative; and (**B**) Gram-positive bacteria.

Bacteria have a number of virulence factors, including adhesins, capsular polysaccharides (CPSs), invasion enzymes (e.g., hyaluronidase, collagenase, and coagulase) and toxins (including exo- and endo-toxins) (Table 1), that allow for successful pathogenesis [6]. Virulence factors facilitate colonisation of the host through initial host cell attachment, host cell entry, immune evasion, replication of the bacterial cell (whether it is intracellular or extracellular) and inhibition of host immune cell functioning such as the inhibition of phagocytosis [7,8,9]. Carbohydrate-based interactions of these virulence factors are crucial in the colonization of the host and, in particular, during adherence to the host’s cells. One such adherence factor is the pilin, which is a filamentous organelle primarily located on the surface of Gram-negative bacteria and, more recently discovered, of Gram-positive bacteria (Figure 1) [10,11]. These pili are composed of proteins arranged in a scaffold-like manner, which are anchored to the bacterial cell surface. Lectins are non-enzymatic proteins that bind to distinct carbohydrate moieties. At the tip of the rod-shaped pilus is a lectin domain that acts as an adherence factor, often referred to as an adhesin, and determines the binding specificity of the pilus [11]. Similar to pili, microbial surface components recognizing adhesive matrix molecules (MSCRAMMs) are also attached to the surface of bacteria. MSCRAMMs are anchored to the cell wall via sortases, initiating adhesion by binding to host extracellular matrix proteins such as collagen, laminin, fibronectin and fibrinogen [12,13]. MSCRAMMs and exopolysaccharide are often involved in biofilm formation, allowing bacterial cells to attach to biotic and abiotic surfaces [14].

**Table 1:** Some virulence factors involved in bacterial-host interactions.

Virulence Factors	Description	Reference
Fimbriae	Thin, thread-like lectins that extrude from both Gram-positive and -negative bacteria.	[15]
Pili	Similar to fimbriae but also involved in bacterial conjugation.	[16]
Biofilm	Exopolysaccharide, bacterial surface proteins amphiphilic molecules and extracellular DNA, allowing bacteria-bacteria/host cell/abiotic attachment.	[14]
S-layer	One or more glycoprotein(s) that coat many Gram-positive and -negative bacteria.	[17]
Capsular polysaccharide (CPS)	Composed of repeating units of oligosaccharides encapsulating Gram-positive and -negative bacteria.	[18]
Lipopolysaccharide (LPS)	Also known as endotoxin. Lipid A and polysaccharide comprised of repeating units of oligosaccharides located on the outer surface membrane in Gram-negative bacteria.	[19]
Teichoic acids	Cell wall components of Gram-positive bacteria, often involved in adherence and biofilm formation.	[20]
Surface proteins	Proteins/glycoproteins found on the bacterial cell surface. Can mediate host cell/extracellular matrix/abiotic surface attachment.	[14]

Bacterial-host carbohydrate interactions can be difficult and time-consuming to investigate, partially because of the complexity of initially deciphering which interactions occur and the difficulty of purification or synthesis of sufficient quantities of complex carbohydrate structures. With advances in microarray technologies, carbohydrate microarrays can provide a sensitive, high-throughput (HTP) platform that allows for the analysis of bacterial carbohydrate mediated interactions in a format that increases the number of experiments possible with limited sample amounts. In 2004, Disney and Seeberger [21] used carbohydrate microarrays for the rapid detection of whole cells of *Escherichia coli.* Since then, the assessment of bacterial interactions using carbohydrate-based microarrays, whether for diagnostics, carbohydrate specificity or potential therapeutic inhibition studies, has thrived. Major developments and variations in labelling techniques, microarray slide surface chemistry and types of carbohydrates printed on these microarrays, ranging from monosaccharides and microbial polysaccharides to mucins [22,23,24], have enabled carbohydrate microarrays to move from proofs of concept to maturing laboratory tools.

Many strains of Gram-negative bacteria express structurally unique polysaccharide structures in the form of capsular polysaccharide (CPS) or the O-specific polysaccharide (O-PS) from their lipopolysaccharides (LPSs) (Figure 1) [25]. LPS is an amphipathic molecule comprised of three parts; lipid A, core oligosaccharide and O-PS, which can exhibit high structural diversity even within the same species, and covers up to 75% of the bacterial cell surface (Figure 1 and Table 1). It plays a major role in pathogenesis and the O-PS is immunogenic [26]. In addition, Gram-positive bacteria, which express CPS, often have structures unique to particular strains. Thus, polysaccharide structures can be pathogen-specific, and these unique structures are the basis of heat resistant serotyping. Demonstration of anti-LPS or anti-CPS antibodies in patients is an indirect indication of infection and is used in the diagnosis of many bacterial infections [26]. Therefore, serum antibody recognition of specific bacterial polysaccharides could be used as a diagnostic for particular infections and multiplexed bacterial polysaccharide microarrays have been proposed as a novel rapid HTP diagnostic approach [3,25,26].

**Figure 2:**
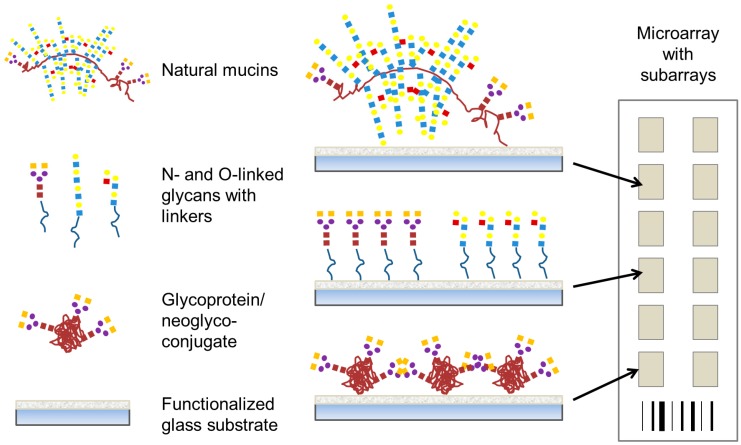
Representative presentation of carbohydrate structures on microarrays to profile bacterial interaction. For this purpose, natural mucins, oligosaccharides with suitable linkers, and glycoproteins or neoglyconjugates may all be covalently attached to microarray surfaces.

In this review, we discuss carbohydrate-based microarrays that have been used to explore bacterial-carbohydrate interactions. These studies are presented in three main sections encompassing three main types of carbohydrate-based microarray platforms; (i) carbohydrate or glycan microarrays (Figure 2), consisting of low molecular mass mono- to oligo-saccharides as found in the host system which have been profiled with whole bacterial cells or recombinantly expressed potential adhesins from bacteria to determine their carbohydrate-binding specificity; (ii) whole mucin microarrays (Figure 2), which were profiled with lectins and whole bacteria to determine mucin glycosylation and bacterial binding tropisms; and (iii) microarrays constructed from bacterial polysaccharides or components of bacterial glycosylation, which have been profiled with serum or antibodies. Secreted bacterial toxins [27,28], fungal cells [29] and fungal extracellular vesicles [30] and recombinantly expressed viral surface proteins [31,32] have also been profiled on carbohydrate microarrays for their specificity. However, these molecules and organisms are outside the scope of this review and will not be discussed here.

## 2. Carbohydrate Microarrays

Slide surface chemistry can vary and impacts on the molecule that is printed and the presentation of that molecule to the microenvironment. Coated and functionalised glass slide surfaces, including aldehyde, nitrocellulose, epoxide, polylysine, hydrogel and maleimide groups, are a popular choice for microarray probe printing. Complex *N*-linked oligosaccharides chemo-enzymatically synthesised *in situ* on the slide surface in nanoscale have also been developed [33]. Immobilisation of the probe on the slide surface also impacts on the presentation and availability. Noncovalent binding of carbohydrate probes on to a microarray surface relies on passive absorption. However, covalent methods of attachment are often preferred over noncovalent immobilisation as the creation of a covalent bond creates a stable linkage between the carbohydrate-containing molecule and the array substrate (Figure 2) [34]. For this covalent method of attachment, carbohydrate ligand presentation also varies depending on the choice of linker [35,36].

Detection of the binding agent using a laser scanner of a fluorescently labelled recognition molecule is the most used method of detecting binding events [36]. As carbohydrate microarrays have been used for a wide range of purposes, labelling techniques differ depending on what is being profiled on the microarray. For assessing bacterial interactions, detection methods have involved indirect labelling of the specific glycoprotein with a fluorescently labelled secondary antibody [3]. However, this method relies on the selection of the glycoprotein in advance to retrieve a suitable antibody for the glycoprotein of interest. Bacteria are also commonly fluorescently labelled using a cell-permeable nucleic acid stain [21], while other methods involve fluorescently labelling bacterial cells with carboxyfluorescein diacetate, succinimidyl ester (CFDA-SE) [37], with the fluorescently labelled bacteria bound to the carbohydrate microarray being detected by a fluorescence microarray scanner.

The use of carbohydrate microarrays to elucidate whole bacterial cell-carbohydrate interactions began to emerge when Disney and Seeberger [21] used a carbohydrate microarray for the detection of *E. coli* in complex biological mixtures in 2004. *E. coli* is a Gram-negative, facultative anaerobic bacterium that usually co-exists with healthy animals. However, various strains of *E. coli* have adapted virulence factors causing disease in otherwise healthy hosts. Adhesins, such as those found in pili, allow pathogenic *E. coli* to colonise areas of the body such as the urethra and the small intestine. This colonisation results in urinary tract infections, often caused by uropathogenic *E. coli* (UPEC), and symptoms such as diarrhoea.

The carbohydrate microarray used by Disney and Seeberger consisted of decreasing concentrations of five monosaccharides, mannose (Man), glucose (Glc), *N*-acetyl-d-glucosamine (GlcNAc), galactose (Gal) and fucose (Fuc), functionalised with reducing end ethanolamine. These monosaccharides were covalently linked to the slide surface coated with homobifunctional disuccinimidyl carbonate linkers via amide bond formation. Two *E. coli* strains were used in this study, *E. coli* ORN 178 and ORN 209, a *fimH* mutant of ORN 178, which encodes for the FimH protein responsible for Man binding. *E. coli* cells were fluorescently labelled with SYTO^®^ 83, a fluorescent nucleic acid stain that internalises into the bacterium and does not interfere with surface presented molecules, and incubated on the printed microarray. The microarray was then scanned and binding of the fluorescently labelled bacterial cells to only the arrayed Man was observed. The microarray permitted the detection of bacterial interactions in a strain-dependent manner, as the FimH mutant strain *E. coli* ORN 209 had a reduced affinity for Man. This carbohydrate microarray proved to be an excellent platform for bacterial detection and also allowed for pathogens to be harvested from the microarray for further culture analysis, which is an advantage over standard PCR analysis to detect pathogens. This microarray platform was also used to measure the ability of Man containing compounds (Man, *p*-nitrophenyl-α-d-mannospyranoside (*p*-NPMan) and a water soluble multivalent Man-functionalised polymer) to inhibit the binding of *E. coli* ORN 178 to the surface-arrayed Man. The inhibition of *E. coli* ORN 178 binding to Man was most effective with the Man-functionalised polymer, followed by p-NPMan and Man, respectively. Significantly, bacteria were detected in complex biological samples containing serum and sheep erythrocytes using this carbohydrate microarray [21].

Pathogenic strains of *E. coli* can be generally categorised into six groups: enteroaggregative *E. coli* (EAEC), diffusely adherent *E. coli* (DAEC), enteropathogenic *E. coli* (EPEC), enterohaemorrhagic *E. coli* (EHEC), enteroinvasive *E. coli* (EIEC) and enterotoxigenic *E. coli* (ETEC). The pyelonephritis-associated (P) pilus is a well characterised virulence factor expressed by UPEC. The P-pilus possesses a lectin domain, known as PapG, conferring adhesion to urinary tract cells using the host-expressed Gal-α-(1→4)-Gal structures [38]. The type l pili are also associated with UPEC, which is encoded by the *fim* gene cluster. On the tip of the type l pili is the adhesion factor, FimH. Type IV pili have been the subject of much research recently. These pili are composed of a single pilin subunit and often have an adhesive subunit located at the tip of the pilus. However, these pili differ in comparison to type I and P pili in that the pili are assembled at the cytoplasmic membrane, rather than the extracellular space. Moreover, type IV pili can retract through bacterial cell walls with the adhesive tip remaining adherent to the receptor. The type IV pili have been associated with *Pseudomonas aeruginosa* and *Vibrio parahaemolyticus* host cell adhesion, with PiIV of the type IV pili contributing to host cell adhesion for *Neisseria* spp. [11].

In 2013, Lonardi *et al.* [39] screened the lectin adhesin domains F17G and FedF, associated with F17 and F18 fimbriae located on ETEC, respectively, and the lectin adhesion domain FimH from the Type-1 fimbriae of a UPEC strain of *E. coli*. *E. coli* uses fimbrial adhesins such as F17G, FedF and FimH to initiate colonisation of the host epithelial lining, allowing septicemic and ETEC strains of *E. coli* to secrete toxins resulting in diarrhoea in young livestock [40]. Different variants of fimbrial adhesins can be associated with different sources of isolation. For example, F17b fimbriae are expressed in an ETEC strain isolated from calves and lambs, F17a in ETEC strains isolated from bovine hosts, while clinical strains of *E. coli* F17G fimbriae have six natural variants (designated F17a–F17f). However, until recently, the effect of the variations of these fimbrial lectin domains on their carbohydrate specificities was not well characterised. Thus, the carbohydrate specificities of the recombinantly expressed lectin domains of these fimbrial adhesins were analysed using carbohydrate microarrays and binding of the recombinant proteins was detected using fluorescently labelled secondary antibodies [39]. Two different carbohydrate microarrays were used in this study: a microarray constructed by the Consortium for Functional Glycomics (CFG) consisting of synthetic and natural carbohydrates, and a shotgun carbohydrate microarray where the glycans used to construct this microarray were isolated from the jejunum of a three-week old piglet (the target tissue of F18-fimbriated *E. coli*). The synthetic and natural carbohydrates were derivatised and covalently attached to amine reactive NHS-activated glass slides, while piglet intestinal glycolipid and *N*-linked oligosaccharides were enzymatically released and directly immobilised onto epoxy-coated slides for the shotgun carbohydrate microarray. Using these microarrays, the terminal non-reducing GlcNAc-β-(1→3)-Gal disaccharide showed consistent binding with the fimbrial variant of F17G. Using the shotgun microarray, the lectin fimbrial variant FedF and F18-fimbriated bacteria were found to bind to the blood group A type 1 hexasaccharide, a glycan that was not on the CFG glycan microarray but was present on the shotgun microarray. Thus, the carbohydrate ligands of fimbrial adhesion variants associated with the adaptation of enterotoxigenic *E. coli* were identified using synthetic and shotgun glycan arrays in parallel, allowing for both the broad (CFG glycan microarray) and more biologically relevant (shotgun approach) screening of glycan receptors for fimbrial adhesins [39].

**Figure 3:**
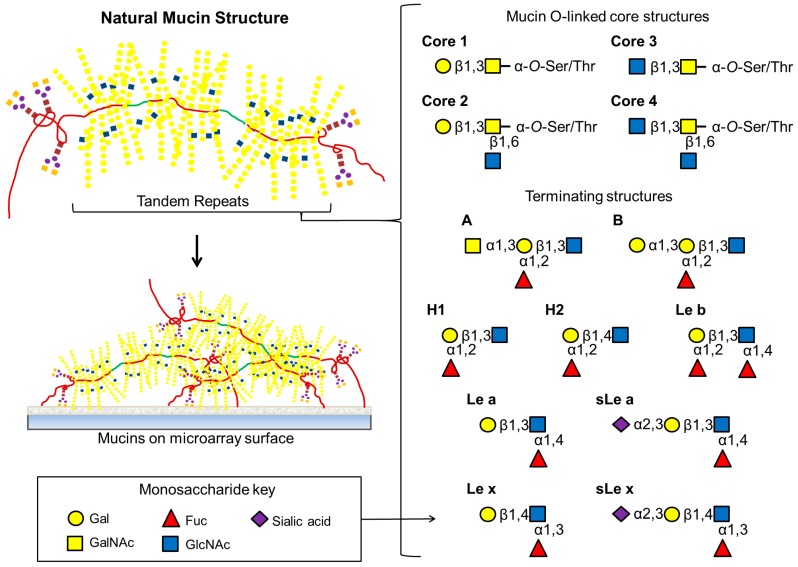
Natural mucins have extensive *O*-linked structures attached to Ser or Thr residues within tandem repeats along with a relatively small number of *N*-linked oligosaccharides at both amino and carboxy ends. As depicted here, the most common mucin core structures are composed entirely of differing combinations of GalNAc, GlcNAc or Gal. Extended *O*-linked oligosaccharide chains are frequently terminated with a variety of Lewis and blood group antigens.

*V. parahaemolyticus* is a Gram-negative pathogen often associated with seafood poisoning and causes acute inflammatory gastroenteritis. *V. parahaemolyticus* utilises type IV pili to adhere to host cell receptors enabling this bacterium to release effector proteins via the type three secretion system into the host cell. This permits successful pathogenesis by enabling a wide variety of cellular processes such as phagocytosis inhibition, inflammation and autophagy induction [34,41]. The mannose-sensitive haemagglutinin (MSHA) pilus, of which the major pilus subunit is encoded by the *mshA* gene, is a type IV pilus expressed by *V. parahaemolyticus*. Previously, it had been shown that the MSHA pilus contributed to the adherence of *V. parahaemolyticus* to abiotic surfaces [42] but carbohydrate ligands for host cell adherence and the role of MshA1, the major type IV pilin subunit, in host pathogenesis was previously unknown. Carbohydrate microarrays consisting of glycoproteins and neoglycoconjugates conjugated via amine groups to a hydrogel surface were used to assess bacterial-carbohydrate interactions associated with the MSHA pilus of *V. parahaemolyticus* [34]. Wildtype *V. parahaemolyticus* and ∆*mshA1* mutant cultures were fluorescently labelled with SYTO^®^ 82 and then directly incubated on the microarray. There was a greater than two-fold decrease in binding for isogenic mutant ∆*mshA1* to several structures such as blood group A and B, lacto-*N*-difucohexaose, lacto-*N*-fucopentaose I, Lewis a (Le^a^), Lewis b (Le^b^) and Lewis x (Le^x^) and asialo-GM1 compared to the wildtype strain (Figure 3). This study confirmed that the MSHA pilus is exploited by *V. parahaemolyticus* for host cell binding, cytotoxicity, cell rounding and the secretion of IL-8. Intense binding of the wildtype strain to certain carbohydrate structures compared to the ∆*mshA1* isogenic mutant suggested a role for these structures in MSHA pilus-mediated *V. parahaemolyticus*-host cell adhesion and pathogenesis [34].

*Campylobacter jejuni* is a microaerophilic, Gram-negative, motile, flagellate, spiral intestinal pathogen and is the leading cause of food borne bacterial gastroenteritis worldwide [43,44]. Chickens harbour *C. jejuni* as a commensal and poultry is an important reservoir of human infections [43]. Infection with *C. jejuni* is the most frequent trigger associated with patients who subsequently develop Guillain-Barré syndrome, a progressive neuromuscular paralysis [44]. The mechanisms leading to commensalism in chickens or pathogenicity in humans are poorly understood. There are many physiological differences between avians and humans but temperature differences, with avian species having a body temperature of 42 °C and humans 37 °C, are a potential signal for host-specific infection [45]. Differential gene transcription has been observed for *C. jejuni* between 42 and 37 °C, including genes encoding proteins involved in membrane structure modification [46], in addition to changes in bacterial cell surface glycosylation [47].

*C. jejuni* strains 11168-GS, a genome sequenced, frequently passaged, poor coloniser, and 11168-*O*, the original of the same strain but an infrequently passaged isolate and a potent coloniser, were profiled on carbohydrate microarrays to investigate the involvement of carbohydrates in host cell adherence [37]. Changes in temperature and oxygen concentration during bacterial culture were assessed to determine whether external growth conditions, similar to mammalian, avian host environments or the external environments, had an effect on glycan-mediated bacterial interactions. Propylamino-glass slides were functionalised with a reactive isocyanate surface by coupling with 1,6-diisocyanatohexane [48]. Carbohydrates were covalently conjugated to the functionalised slides catalysed by pyridine vapour. *C. jejuni* strains 11168-GS and 11168-*O* were fluorescently labelled with the fixable-cell-permeant CFDA-SE, which enters the cell and is cleaved by intracellular esterases to form the amine reactive fluorescein-based label CFSE. This, in turn, conjugates to available amine groups and gives the cell its fluorescence. The labelled bacteria were incubated on the microarray at 42 and 37 °C, and fluorescence intensities were measured using a microarray scanner. At growth conditions of 25, 37 and 42 °C, both strains bound to Man, Fuc, Gal and sialic acid (Neu5Ac), which was verified using Caco-2 adherence assays with a pre-treatment of Caco-2 cells with a competing lectin. These competing lectins included Gal-β-(1→4)-GlcNAc (type II LacNAc)-specific *Erythrina cristagalli* agglutinin (ECA), sialic acid-specific *Limax flavus* agglutinin (LFA), Man-specific concanavalin A (Con A), α-(1→2)-linked Fuc-specific *Ulex europaeus* agglutinin I (UEA-I), α-(2→3)-linked sialic acid-specific *Maackia amurensis* agglutinin (MAA) and α-(2→6)-linked sialic acid-specific *Sambucus nigra* agglutinin (SNA). Bacterial adherence was represented as a percentage of bound bacteria in the absence of a competing lectin. Interestingly, various growth conditions did not affect the ability of *C. jejuni* 11168-GS to bind to different Man-containing structures, while the *C. jejuni* strain 11168-*O* only bound to Man after culturing at 25 °C with normal oxygen conditions. As many pathogenic bacteria use the Man-specific FimH lectin for attachment and adherence to the host tissue, carbohydrate analysis of Man-binding bacteria can provide insight into bacterial-carbohydrate interactions occurring in the host, which may be implicated in pathogenesis. When grown under avian and mammalian temperatures and different oxygen conditions, *C. jejuni* strains bound to fucosylated structures, such as the Le antigens that are commonly found on GIT mucins and epithelial cell surfaces [49]. This carbohydrate microarray provided an important understanding into the carbohydrate structures involved in *C. jejuni* adhesion of host tissue [37].

Additional *C. jejuni* isolates from humans and chickens were assessed for carbohydrate interactions using the same carbohydrate microarrays as described above by the same group [50]. All isolated *C. jejuni* strains bound to carbohydrate structures with terminal Gal and fucosylation, which was confirmed by cell binding inhibition assays with UEA-I. It was suggested that this broad range of Gal recognition and low specificity for fucosylated structures may help to explain why *C. jejuni* has such a broad range of hosts, from mammalian to avian. Interestingly, binding to Man structures appeared to be greatest when the cell was under environmental stress (normal oxygen and room temperature) but was eradicated when the bacteria were grown at 37 or 42 °C under microaerobic conditions, suggesting that this bacterial interaction may be important for initial pathogen-host interactions while Gal and Fuc binding may be required for the persistence of the infection inside the host [50]. Supporting previous research [37,51,52], initial attachment of *C. jejuni* may occur by binding to sialylated and mannosylated structures commonly found on the human glycoprotein MUC1 in the human gastrointestinal mucosa. In addition, preferential binding of *C. jejuni* to Fuc and Gal in mammalian and avian-like environments may allow for this bacteria to persist in intestinal epithelium crypts by binding to the heavily fucosylated and galactosylated mucins, for example MUC2 [37,50,51,52].

*Helicobacter pylori* is a microaerophilic, Gram-negative, motile, flagellate, spiral gastric pathogen and is present in almost half of the world’s population [53]. It colonises the gastric mucus of humans and some other primates, but does not naturally infect other hosts, and causes gastritis, peptic ulcer disease, mucosa-associated lymphoid tissue (MALT) and gastric adenocarcinoma [43,54,55]. It adheres to the cell surface and the mucus using several adhesins; the blood group antigen binding adhesin (BabA), which binds to the Le^b^ motif, and the sialic acid binding adhesin (SabA), which binds to the sialyl Le^x^ (sLe^x^) and sialyl Le^a^ (sLe^a^) motifs (Figure 3) on glycoproteins and glycolipids. During infection and inflammation, expression of the sialylated Le structures are increased in the gastric mucosa [54] and the mucus layer acts as a reservoir for the pathogens [43].

In 2005, the carbohydrate specificities associated with the *H. pylori* adhesins BabA and SabA were investigated using a glycoprotein and neoglycoprotein array [56]. Here, glycoproteins or neoglycoproteins were spotted onto nitrocellulose membranes and enzymatically pretreated. Fluorescein-5-isothiocyanate (FITC) was used to fluorescently label the *H. pylori* isolates, which were incubated on the array and the fluorescence of adherent bacteria was measured using a laser scanner. Using wild type *H. pylori* and ∆*sabA* and ∆*babA* mutants, it was confirmed that the BabA adhesin interacts with Le^b^ and other fucosylated antigens, while *H. pylori*-laminin binding is mediated by SabA [56]. Moreover, binding of *H. pylori* to the glycoproteins fibronectin and lactoferrin was not mediated by either SabA or BabA, as a *sabA/babA* double mutant maintained binding to these glycoproteins on the carbohydrate array. Furthermore, *H. pylori* binding to the human salivary mucin MUC5B was mainly attributed to BabA, and SabA to a lesser degree. A ∆*sabA/*∆*babA* double mutant abolished binding to MUC5B, which indicated the importance of Le^b^, fucosylated antigens and sialic acid-containing carbohydrate motifs in *H. pylori* binding [56].

## 3. Mucin Microarrays

Mucus coats the epithelial surfaces inside the human body in the gastrointestinal, respiratory, urinary and reproductive tracts and the cornea of the eye. It hydrates mucosal surfaces, protects from chemical and biological insult and mechanical abrasion and has a role in many more specific biological processes including the innate and adaptive immune system response. The mucus layer is comprised of a combination of complex molecules, with mucins as the major component. Mucins are a family of high molecular mass glycoproteins encoded by a family of over 20 MUC genes [4,57,58]. They can be membrane-bound or secreted, with secreted mucins forming the bulk of the macromolecular molecules of the epithelial surface mucus layer. Mucins are densely substituted with mucin-type *O*-linked oligosaccharides and occasionally *N*-linked oligosaccharides, and carbohydrates comprise 50%–90% of the molecule by mass. Eight core structures have been elucidated for mucin-type oligosaccharides *O*-linked to serine or threonine via an *N*-acetylgalactosamine (GalNAc) residue (Figure 3) [57,59,60]. These structures can be extended by the residues GalNAc, GlcNAc, Gal and Fuc and the oligosaccharides can be terminated or substituted with sialic acid or sulfate groups. These structures can be very heterogenous, even on the same molecule, with the oligosaccharides themselves varying in chain length, residue composition, residue linkages, branching and the degree of substitution of terminal and peripheral residues and groups [36,60]. These structures provide ligands for cell adhesion and mammalian lectins amongst other biological functions, including participation in immune system control, and specific functions are related to specific structures [60]. Mucin protein expression and glycosylation is site- and tissue-specific and mucin glycosylation varies dynamically with health and disease status and during growth, development, infection, cell differentiation and activation [4,57,60].

Mucin glycosylation also provides the main source of ligands in the mucus layer for bacterial adhesion *via* adhesins, which selectively recognise and bind to specific carbohydrate structures. Accordingly, certain bacterial populations are associated with particular regions *in vivo* [4,7,57,61]. Bacteria and mucus have been shown to engage in mutually beneficial “cross-talk” which influences epithelial cell differentiation in the postnatal intestine and contributes to host nutrition and host defence against pathogenic strains by competition for available nutrients and microenvironment occupation. Mucins also function as a decoy for pathogen or toxin clearance; the pathogen or toxin binds to the mucin and is cleared from the mucosal layer with the mucin in normal mucus turnover. Pathogens may also exploit mucin glycosylation to facilitate colonisation and infection. Further, when the mucosa is damaged, commensal bacteria can become opportunistic pathogens [4].

As well as the variation in oligosaccharide structures themselves, mucin glycosylation can also vary in the density of substitution of the oligosaccharides along the protein backbone, the distribution of specific structures and their presentation in space, all of which directly impact on their biological function and bacterial binding ability [36,57,62]. Elucidating the interactions of mucins with bacteria can be difficult as the yield of purified mucin from biological samples is often very low and techniques such as isothermal calorimetry, direct binding assays in microtitre plate or blot format, surface plasmon resonance or inhibition assays using immobilised mucin or cells are not favourable to HTP analysis, and consume substantial quantities of reagents including the purified mucin [36,62]. In addition, structural analysis of individual *O*-linked oligosaccharides on mucins is difficult due to their significant structural heterogeneity and the lack of a universal enzyme for release of any *O*-linked oligosaccharide structures [59]. Often conventional carbohydrate microarrays (described in Section 2) cannot provide a true picture of the binding of carbohydrate binding proteins or whole bacteria in a biological context, as only single structures are presented in a non-biologically relevant fashion due to the technical constraints of the platform [63]. Molecules with multivalent and three-dimensional presentations of carbohydrates, such as natural mucins, are required to study density effects, avidity and interactions dependent on concurrent binding with several different structures [36,62].

A natural mucin microarray consisting of 35 mucins purified from mucosal surfaces in different animals and 2 mucins purified from human gastrointestinal tract (GIT) cell lines (LS174T and E12) was constructed for the first time to facilitate the profiling of mucin glycosylation and screening of bacterial interactions with the arrayed mucins in a HTP format, while consuming miniscule quantities of purified mucin [36]. The animal mucins were mainly purified from the GIT, but the respiratory and reproductive tracts were also represented. As the mucin protein backbone termini are less densely substituted with *O*-linked oligosaccharides, these areas are more available for conjugation strategies. Thus, the purified mucins were conjugated to an NHS functionalised hydrogel surface via their amine groups to allow optimal presentation and accessibility of the oligosaccharides on the intact whole mucins (Figure 3). The three dimensional hydrogel surface of the microarray offers low background and negates the need for a separate blocking step [36], which is advantageous when profiling whole bacteria on a surface as they may adhere non-specifically to “sticky” molecules such as bovine serum albumin commonly used for blocking. The mucins were profiled on the microarray with a panel of lectins to decipher their glycosylation and mucin glycosylation was found to vary by species and originating location in the body [36].

This natural mucin microarray was subsequently employed to elucidate the mechanisms of interaction of *H. pylori* and *C. jejuni* with mucus and mucins [43]. These mucosal pathogens infect different niches in the human GIT and are closely related phylogenetically. Adherence to carbohydrate ligands is known to mediate *H. pylori* attributed disease [54]. Four strains of *H. pylori* (strains 26695, J99 and G27 and the babA knockout mutant of G27 (G27∆babA)) and six strains of *C. jejuni* (strains 81–176 and 11168, chicken isolates CC18 and CC19, and human isolates H1 and H3) were stained with SYTO^®^ 82 and were profiled for binding interactions on the natural mucin microarray. Despite their close phylogenetic relationship, the strains of *C. jejuni* and *H. pylori* bound to distinct sets of mucins. The *C. jejuni* strains most intensely bound to the mucins from chicken cecum, proximal small intestine and large intestine with varying intensities, with greatest binding to chicken large intestine mucin and least to chicken cecum mucin, and there were no statistically significant differences in binding intensities between 37 and 42 °C [43]. The binding of *C. jejuni* to chicken mucins on the mucin microarray concurred with a previous report of a carbohydrate-mediated attenuation of binding and internalisation of *C. jejuni* in to the HCT-8 human epithelial intestinal adenocarcinoma cell line in the presence of these chicken mucins. The greatest attenuation was observed with chicken large intestine mucin (1500-fold reduction) and least attenuation with mucin from chicken cecum (5-fold reduction) [64]. Interestingly, differential glycosylation profiles were elucidated for these three mucins using lectin profiling on the natural mucin microarray and the proportionally greater presence of terminal GalNAc and/ or sulfated GalNAc on the mucin from chicken large intestine was suggested as the favoured binding motif of *C. jejuni* [36]. The *H. pylori* strains bound to several mucins including porcine stomach mucin but at a lower intensity than *C. jejuni* bound to the chicken mucins.

The natural mucin microarray was also used to profile the interactions of the commensal GIT strains *Lactobacillus salivarius* AH102 and *Bifidobacteria longum* AH1205 in a similar manner to that described above [22]. These two commensal strains had previously been shown to attenuate the association and internalisation of *C. jejuni* in to E12 cells [65]. When profiled on the natural mucin microarray, they both interacted with a subset of mucins but to varying degrees, *i.e.*, the pattern of their interaction was different. Their binding tropisms were not related to species or originating location of the mucins, which indicated the importance of the specific mucin glycosylation [22].

Artificial glycopolymers mimicking mucins have been synthesised by the Bertozzi group and consist of a functionalised polymer backbone with substitution of aminoxy functionalised carbohydrates along the backbone at controlled distances and custom functional groups at the termini of the polymer backbone to facilitate surface conjugations and attachment of various labels [63]. Aberrantly glycosylated mucins are overexpressed in tumours, and mucins from certain tumour types display α-linked GalNAc only (Tn antigen) [66]. Synthetic mucins displaying α-linked GalNAc with varying valencies (68–170 or 17) and labelled with Cy3 at one terminus and biotin at the other were contact printed on to a streptavidin coated glass slide at either three concentrations (75–400 nM for valencies 68–170) or five concentrations (75–1200 nM for the valency 17 polymer) to construct a microarray presenting the synthetic mucins with varying valencies and molecular spacing. Four AlexaFluor^®^ 647 (AF647) labelled lectins with binding affinities for *a*-linked GalNAc, soybean agglutinin (SBA), *Wisteria floribunda* lectin (WFL), *Vicia villosa* agglutinin B4 isolectin (VVA-B4), and *Helix pomatia* agglutinin (HPA), were evaluated on the microarray for their ability to cross-link the synthetic mucins using fluorescence resonance energy transfer (FRET) produced upon lectin binding between the Cy3 and AF647 labels [62]. Valency-dependent binding of three out of the four lectins was observed (SBA, WFL and VVA). All four lectins cross-linked the low-valency synthetic mucins while only SBA cross-linked the high-valency polymers demonstrating the importance of spacing, density and presentation of the carbohydrate components for lectin binding [62]. Although it has not been employed to study bacterial interactions to date, once particular carbohydrate structures from mucins that interact with bacteria are deduced, these synthetic mucins in a microarray format could be used to elucidate the influence of spacing, density and avidity in bacterial-carbohydrate interactions.

More recently, a human colonic mucin microarray was constructed with colonic mucin from patients with colon cancer (healthy tissue away from the tumour site; control, *n* = 7) and ulcerative colitis (UC, *n* = 5) in a similar manner to the natural mucin microarray [36] and was used to profile the interactions of the commensal microbes *Akkermansia muciniphila* and *Desulfovibrio* spp. [67]. UC is associated with altered mucin composition including decreased sulfation, a decreased abundance of *A. muciniphila* and an increased load of *Desulfovibrio* spp. [68,69,70]. *A. muciniphila* is a Gram-negative, non-motile anaerobe which degrades mucin [71] and *Desulfovibrio* spp. are Gram-negative, motile anaerobes (with some species of the genus able to grow in the presence of oxygen) which reduce sulfate [72] and metabolise sulfate on sulfated mucins in the intestinal tract [67]. Both species colonise the mucus layer of the human colon but their ability to bind to colonic mucin and whether this binding was affected by the changes in UC colonic mucin was previously unknown. The reference strains *A. muciniphila* ATCC BAA-835 and *Desulfovibrio desulfuricans* ATCC 27774 and the clinical isolates of *A. muciniphila* and *Desulfovibrio* spp. from one control patient and three patients with active UC were profiled on the human colonic mucin microarray. Both *A. muciniphila* and *Desulfovibrio* spp. bound to control and UC colonic mucins. Both reference and clinical isolates of *A. muciniphila*, *Desulfovibrio desulfuricans* ATCC 27774 and one clinical isolate of *Desulfovibrio* spp. had increased binding to UC colonic mucin compared to control colonic mucin while the other two clinical isolates of *Desulfovibrio* spp. showed no difference in binding between UC and control colonic mucins. This work provided the first evidence of direct binding of these commensals to colonic mucins and altered binding between UC and control colonic mucins [67].

## 4. Microbial Polysaccharide and Carbohydrate Microarrays

In 2002, Wang and colleagues printed fluorescein isothiocyanate (FITC)-conjugated dextrans of different molecular masses along with inulin onto nitrocellulose-coated glass slides to verify that polysaccharides could be immobilised non-covalently on a surface and remain there, even after extensive washing [3]. The larger dextran molecules were found to have been better retained than the smaller molecular mass dextrans but all were still detectable. Non-labelled dextrans from the genera *Leuconostoc* and *Streptococcus* and the family Lactobaccillaceae were then immobilised on the microarray surface. Dextrans from different species vary in their linkages and molecular masses. Many dextrans are typically linear polymers of Glc*p* with α-(1→6)-linked backbones but may also have branched structures with several glycosidic linkages of α-(1→6), α-(1→3) and α-(1→2) which may be individually detected by specific antibodies. Anti-dextran antibodies were incubated on the dextran microarray after blocking with 1% bovine serum albumin (BSA) in PBS and antibody binding was detected with a labelled secondary antibody. Because the appropriate anti-dextran antibodies identified their respective specific immobilised structures, this confirmed that the polysaccharide structures were preserved and accessible after immobilisation on the nitrocellulose surface [3].

The group then extended the array to encompass 48 carbohydrate-containing macromolecules, which included polysaccharides, and semi-synthetic glycoconjugates. Several microbial pathogens were represented, including *Klebsiella*, *E. coli*, *Pneumococcus*, *Meningococcus* and *Haemophilus influenzae* type A, but the purification methods or nature of the polysaccharides from these sources that were printed were not detailed. Human serum IgM and IgG antibodies from 20 normal volunteers were profiled to reveal distinct anti-carbohydrate specificities of the antibodies with IgM showing mainly specificity for the *Klebsiella* polysaccharides and the IgG anti-carbohydrates had much broader binding affinities [3]. Interestingly, Gram-negative *Klebsiella* spp. are normal human residents, colonising mucosal surfaces but can act as opportunistic pathogens and cause pneumonia, urinary tract infections and neonatal sepsis [73].

LPS microarrays were constructed using LPS purified from *Francisella tularensis* subspecies *tularensis*, *E. coli* strains O111:B4, O26:B6 and O157, *Klebsiella pneumoniae*, *Pseudomonas aeruginosa* serotype 10, *Salmonella enteritidis*, *S. typhimurium* and *Shigella flexneri* serotype 1A printed on nitrocellulose-coated glass microarray slides. Initially FITC-labelled *E. coli* O111 was printed to verify that a concentration-dependent signal was produced and that this signal did not significantly decrease upon repeated washing. Monoclonal antibodies of IgG and IgM isotypes specific for four of the printed unlabelled LPSs were incubated on the microarray, after blocking the slides with 2% BSA in PBS, to verify stable immobilisation of the LPSs and the retained immunogenic structure of the molecules after immobilisation. The lower detection limit was established as 10 ng/mL of the antibody on the microarray platform, which was 100 times more sensitive than the 1 μg/mL required for the more conventional microtitre plate-based immunofluorescence assay. Five samples of tularemia-positive canine serum and two tularemia-negative canine serum samples were incubated on the microarray and all five tularemia-positive sera bound to the *F. tularensis* LPS, but none of the tularemia-negative samples did. Interestingly, two tularemia-positive and two tularemia-negative sera reacted slightly with the *E. coli* 026 LPS and one tularemia-positive sample had moderate binding with the *K. pneumonia* and *E. coli* O111 LPSs, showing evidence of previous infections of these animals [26].

**Figure 4:**
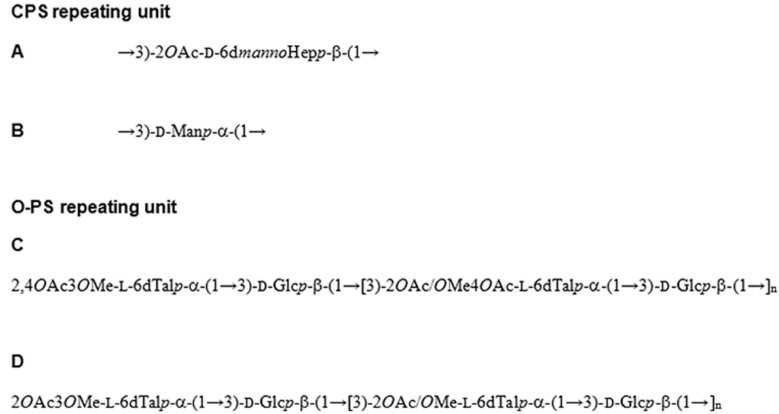
The CPS of O-PS-deficient strains *Burkholderia mallei* BM1987 and *B. pseudomallei* BP2683 consist of a mixture of structures (**A**) and (**B**). *B. mallei* BM1987 has a distribution of 48:52 A:B while structure (**B**) comprises only 7% of *B. pseudomallei* BP2683 CPS [77]. The O-PS structure of *B. pseudomallei* RR2808 (a derivative of strain 1026b) is depicted in structure; (**C**) and is identical to the O-PS of the non-pathogenic *B. thailandensis* strain E264. Structure (**D**) is the O-PS of *B. mallei* BM2308 (a derivative of ATCC 23344) [76].

*Burkholderia mallei* is a Gram-negative, non-motile, facultative intracellular bacterium that causes glanders, a disease characterised by ulcerative nasal and/or tracheal lesions, pulmonary involvement and visceral abscesses. Humans can acquire this disease, but it primarily affects horses and can be contracted by other mammals including dogs and cats [74]. *B. pseudomallei,* a Gram-negative, motile, intracellular pathogen, causes melioidosis in humans and animals. Melioidiosis is responsible for sepsis, which is associated with bacterial dissemination, with lungs as the most commonly affected organ [75]. Diagnosis and treatment of glanders and melioidosis is difficult and melioidosis mortality is approximately 20%–50%, even with treatment [74,75]. *B. pseudomallei* and *B. mallei* can be spread via aerosol and are listed as a Tier 1 select agents by the United States (U.S.) Centers for Disease Control and Prevention (CDC) and as Category B Priority Pathogens by the U.S. National Institute of Allergy and Infectious Diseases [74,76]. Currently there are no human or veterinary vaccines available for *B. mallei* or *B. pseudomallei* [74,75,76]. Both *B. pseudomallei* and *B. mallei* express CPS as a virulence factor, which is essential for survival in animal infection models and aids in host immune evasion (Figure 4) [74,75]. The O-PS components of *B. pseudomallei* and *B. mallei* LPS are also virulence factors and protective antigens [76] and components of CPS and O-PS have been suggested and evaluated as vaccine candidates [74,75,76].

Recently, a polysaccharide microarray was constructed by immobilising *B. pseudomallei* and *B. mallei* O-PS and CPS molecules. A mixture of CPS and LPS was extracted from *B. pseudomallei* strains 1026b, 576 and SRM117 and *B. mallei* strain ATCC 23344, the mixture was hydrolysed to release lipid A and the remaining CPS and O-PS polysaccharides were used for microarray production. All strains produce a common CPS but *B. pseudomallei* 576 has an atypical LPS O-antigen, and *B. pseudomallei* SRM117 is an LPS rough mutant (*i.e.*, it lacks O-PS) (Figure 4) [25]. *B. pseudomallei* and *B. mallei* appear to produce only a limited range of O-PS structures unlike most Gram-negative pathogens [76], which could be an advantage for vaccine and diagnostics development. The polysaccharides were derivatised to glycosylamines by reductive amination and conjugated to an expoxide funtionalised glass surface at six different dilutions with triplicate features for each strain. Inulin was included as a negative control. The polysaccharide microarray was then blocked with 2% BSA and incubated with polyclonal serum from a rabbit immunised with *B. pseudomallei* CPS. Antibody binding to the immobilised polysaccharides was detected by incubation with Cy3- or Cy5-labelled anti-rabbit antibodies. Polysaccharides from all Burkholderia strains were detected by the antibodies with no recognition of inulin, as expected, although the sensitivity for *B. pseudomallei* 576 recognition was markedly less than the rest of the included polysaccharides. Similarly, serum from a human patient infected with *B. mallei* was incubated on the polysaccharide microarray and demonstrated antibody reactivity for all strains [25]. This microarray was also used to demonstrate that antibodies against these polysaccharide antigens were present in melioidosis patient sera but not in non-melioidosis infected human volunteers, which demonstrates the potential of this format to be used for accurate human serodiagnostics [78].

In an expansion of this work, the same group added carbohydrate antigens from the pathogens *Bacillus anthracis* and *F. tularensis* to their microarray to make a multivalent device for detection of all three biothreat bacterial infections [79]. *Bacillus anthracis* is a Gram-positive, spore-forming bacterium that causes anthrax in humans while the Gram-negative *F. tularensis* causes tularemia. A collagen-like glycoprotein, BclA, is the major component of the hairs on the exosporium, the loose-fitting outer surface layer of *Bacillus anthracis* spores. BclA is highly immunogenic and contains two *O*-linked oligosaccharides, a tetrasaccharide and a disaccharide. The non-reducing terminal residue of the tetrasaccharide is 4,6-dideoxy-4-(3-hydroxy-3-methylbutamido)-2-*O*-methyl-d-Glc*p* or anthrose (Ant), which is exclusively found in *Bacillus anthracis* [80]. Both the monosaccharide, Ant, and the *Bacillus anthracis* tetrasaccharide, Ant-β-(1→3)-l-Rha*p*-α-(1→3)-l-Rha*p*-α-(1→2)-l-Rha*p*, were synthesised with linkers terminating in an amino group to facilitate conjugation to the microarray surface. The trisaccharide, d-Gal*p*ANAc-α-(1→4)-d-Gal*p*ANAc-α-(1→3)-d-QuiNAc, where QuiNAc is 2-acetamido-2,6-dideoxy-d-Glc*p*, is found in the repeating unit of *F. tularensis* strain 15 O-PS. This trisaccharide was synthesised and converted to the glycosylamine derivative by reductive amination in ammonium acetate and was printed on the microarray [79].

The multivalent microarray was blocked with 1% casein in PBS and then probed with serum from nine human melioidosis patients and four human non-melioidosis controls, four anthrax-infected rabbits and four control rabbits and four tularemia-infected rabbits and two control rabbits [79]. The human melioidosis serum samples interacted strongly with the *B. pseudomallei* strains while the non-melioidosis samples did not. Similarly, the anthrax-infected rabbit sera interacted with both the Ant and Ant-containing tetrasaccharide with no interaction for the control sera, which indicated that the Ant residue was the immunological determinant. Finally, the serum from the tularemia-infected rabbits interacted with the *F. tularensis* trisaccharide while the control serum did not [79].

A library of synthetic saccharides, which included fragments of the BclA oligosaccharides from *Bacillus anthracis* as well as a number of control carbohydrates, were covalently immobilised on to a photoactive microarray surface. The glass microarray slide was coated with a self-assembled mixed monolayer with photoactive phthalimide chromophores. After printing of the native carbohydrates and exposure to UV radiation, the phthalimide groups covalently link to the carbohydrates by hydrogen abstraction followed by radical recombination. This system offers the advantages of size independence of immobilisation and removes the necessity to derivatise the carbohydrates themselves. Pooled rabbit polyclonal IgG antibodies recognizing anthrax spores were profiled on this microarray, and the degree of IgG binding to the various Ant-containing structures was related to their size, with greatest binding intensity with the tetrasaccharide and least with the monosaccharide. Binding inhibition studies using the Ant monosaccharide and Ant-containing di-, tri- and tetra-saccharides to inhibit the binding of the IgGs were also undertaken on the microarray platform. The Ant monosaccharide was found to be a potent inhibitor in addition to the Ant-containing oligosaccharides. Therefore, the HTP approach of screening large numbers of carbohydrate structures for their antigenic potentials may facilitate the identification of key immunogenic structures of pathogens for vaccine development [77].

The Gram-negative *Salmonella* spp. cause various diseases in humans and animals. Most *Salmonella* species are associated with gastroenteritis in humans but severe disease is associated with *S. enterica* serovars Paratyphi, Cholerasuis and Typhi. There are approximately 1.3 billion salmonellosis infections annually in the human population with 3 million deaths. A *Salmonella* O-PS antigen microarray was assembled by covalently linking synthetic oligosaccharides from O-PSs of *S. enterica* sv. Paratyphi (group A), Typhimurium (group B) and Enteritidis (group D), as well as the full O-PS from *S. enterica* sv. Typhimurium, to linkers with free amine groups. The O-PS-linker conjugates were then printed on an NHS-functionalised glass microarray surface. Saturated surface immobilisation was verified by the fluorescently-labelled GlcNAc-specific lectin *Griffonia simplicifolia* (GS-II) for two of the structures while probing with rabbit antisera directed against *Salmonella* serogroups verified the presence of the rest of the structures and demonstrated some cross reactivity based on structure. A mouse monoclonal antibody with specificity for *S.* Typhimurium group B bound strongly only with larger O-PS fragments [81], in agreement with previous observations that antibodies from larger mammals including humans can react with smaller saccharide antigen epitopes but mouse antibodies generally only recognise larger saccharides [82]. The sera from 10 patients with culture-verified salmonellosis (5 from *S. enterica* sv. Enteritidis (serogroup DO) and 5 from *S. enterica* sv. Typhimurium (serogroup BO)) along with 5 healthy controls were incubated on the microarray. Antibodies were correctly detected (i.e. the bound to the expected O-PS fragment) and high serogroup specificity was observed with the disaccharide antigens. Common O-PS backbone antigens were also detected. Interestingly, one out of the five control sera showed a slightly elevated response to the group BO antigen disaccharide, most likely indicating a previous infection during the individual’s lifetime [81].

More recent development has seen a microbial polysaccharide microarray constructed of the largest library to date, consisting of approximately 300 purified and characterised LPSs and CPSs from a variety of Gram-positive and -negative bacteria [24]. To initially verify the printing of the isolated bacterial polysaccharides, those from *Pseudomonas aeruginosa* O2 were printed on amine-reactive NHS functionalised glass microarray slides. Successful immobilisation and a concentration-dependent response was confirmed by incubation of the microarray with anti-*Ps. aeruginosa* O2 antisera, with a saturation concentration of the polysaccharide established at approximately 125 μg/mL. Pooled serum IgG from five healthy human volunteers showed a distinct pattern of interaction on the microarray and IgM reactivity which was distinct from IgG was also detected. A commercial preparation of intravenous immunoglobulin (IVIG), which is a pooled IgG preparation from over 10,000 individuals, had a similar binding pattern to the human IgG. Serum IgG from mouse and rabbit exhibited distinct patterns of interaction with the microarrays from each other and from the human IgG. Sera from rabbits inoculated with specific microbes were also profiled on this microarray and showed high titre seroreactivity to the individual structure from the inoculated microbe. The innate immune galectins 3, 4 and 8 were also profiled on this microarray and recognised only one structure, from *Providencia alcalifaciens* O5. Subsequent binding experiments using the galectins with *P. alcalifaciens* resulted in decreased viability of the bacteria [24].

Traditional serodiagnosis of certain diseases, including melioidosis and tularemia, is carried out by agglutination assays or variants of these. Typically, these tests use filtered bacterial culture supernatants or crude bacteria and very high dilutions of serum. These current agglutination-type tests can lack specificity for the pathogenic strains themselves and often have high background which can lead to false diagnosis [78]. ELISA diagnostic tests, such as those currently used for salmonellosis diagnosis, rely on the use of crude CPS or LPS preparations. These preparations may be quite heterogenous and exhibit large batch-to-batch variations as no analysis is performed [81]. Thus, a multivalent platform consisting of strain- or species-specific bacterial oligo- or poly-saccharides such as those described above provides a very promising alternative for disease diagnosis and rapid identification of the infecting pathogen. Further, these platforms can also help identify the immunogenic epitope within larger structures, which could prove useful for identifying targets for effective vaccine development.

## 5. Concluding Remarks

Carbohydrate-based microarray platforms have been an underused tool for screening bacterial interactions with specific carbohydrate structures, but they have been gaining popularity in recent years. The use of carbohydrate-based microarrays allows a valuable insight in to the molecular mechanisms of carbohydrate-mediated interactions in pathogenesis and host response prior to subsequent focused investigation, as exemplified by the galectin interactions for *Ps. aeruginosa* discovered using a microbial polysaccharide microarray [24]. Multiplexed bacterial carbohydrate microarrays can diagnose infections not only of the species causing the disease, but potentially the strain also. Use of these microarrays with their defined antigens can help eliminate false positives from more conventional agglutination tests, aid in epidemiology studies, help determine targeted treatments for infection and in identifying immunogenic targets for vaccines.

The development of new microarray platforms such as natural mucin microarrays helps overcome technical difficulties associated with assessing the interactions between pathogenic and commensal bacteria and the mucosal barrier *in vivo*. Determining the nature of the interactions between bacteria and host could help clarify the mechanisms of immune response and lead to new strategies to boost therapeutic treatments.
